# Microbicides 2008 conference: From discovery to advocacy

**DOI:** 10.1186/1742-6405-5-19

**Published:** 2008-08-15

**Authors:** Gita Ramjee, Gustavo F Doncel, Sanjay Mehendale, Elizabeth E Tolley, Kim Dickson

**Affiliations:** 1 HIV Prevention Research Unit, Medical Research Council, Durban, South Africa; 2 CONRAD, Eastern Virginia Medical School, Norfolk, Virginia, USA; 3 Department of Epidemiology, National Aids Research Institute, Pune, India; 4 Family Health International, Durham, USA; 5 HTM/HIV Prevention in the Health Section, World Health Organisation, Geneva, Switzerland

## Abstract

Recently revised statistics show the number of individuals living with HIV at over 33 million worldwide, with 68% being in sub-Saharan Africa. Current HIV prevention methods, such as condom use, monogamy and abstinence, are not always feasible. The need for improved HIV preventative technologies remains urgent. Of these, microbicides represent a promising female-initiated preventative method. Microbicides are designed to be applied vaginally to prevent HIV and STI acquisition. Research is also being undertaken to assess the safety of the product during rectal application.

The biannual Microbicides conference took place in New Delhi, India from 24–27 February 2008. The conference was open to delegates from the scientific and medical fields, as well as communities and advocates. In addition to microbicide research and development, the conference afforded the opportunity for the discussion of key issues such as ethics, acceptability, access, and community involvement.

In this conference report we provide brief summaries of recent advancements made and challenges experienced in microbicide research and development, including updates on basic and clinical science, social and behavioural science, and community mobilisation and advocacy activities pertaining to clinical trials.

## Background

Recent statistics by UNAIDS suggest that 2.5 million (Range: 1.8–4.1 million) people were newly infected with HIV in 2007. About 15.4 million (Range: 13.9–16.6 million) women were living with HIV [1]. The disproportionately higher number of infections among women requires a commitment to address the urgent need for HIV prevention tools. Microbicides are products that are currently in development for use by women with or without their partners' knowledge in order to reduce the growing rates of new infections among women. The microbicide research community spans across a multidisciplinary team of basic and clinical scientists, social and behavioural experts together with committed members of community and advocacy groups who are dedicated to finding a women initiated HIV prevention option.

In February 2008, the Microbicide 2008 (M2008) conference was held in the Asian Sub-continent for first time. Held in New Delhi India, the conference attracted over 1000 delegates from the USA, Europe, Asia, Australia, South America and Africa. This was an important meeting, given the recent developments in the field with several large scale efficacy trials having disappointing outcomes [2-4].

We report on the proceedings of the Microbicides 2008 International conference, with respect to outcomes and discussions from Track A (basic science), Track B (clinical science), Track C (social and behavioural science) and Track D (community and advocacy).

### Track A – Basic science and preclinical development

#### Gustavo F. Doncel

Within Track A speakers updated a wide variety of topics ranging from HIV entry in cervicovaginal epithelium to delivery of microbicides by controlled-release devices. In order to facilitate their description, the highlighted presentations have been divided into four areas: sexual transmission of HIV; emerging candidates; biomarkers, models and preclinical evaluation; and formulation and delivery systems.

#### Sexual transmission of HIV

In his plenary lecture, Tom Hope showed that using cervical explants, photoactivable green fluorescent protein-labeled virus and high-resolution confocal microscopy, HIV was seen to penetrate intact epithelium, predominantly through the interstitium. Although some virions reached the innermost layers of the epithelium, most of them were concentrated in the first 40 microns. This distance was enough to put the virus in contact with cellular targets such as Langerhan's cells and intraepithelial lymphocytes. Similar in vivo experiments in macaques support the conclusion that HIV is able to penetrate the genital mucosa through an apparently intact squamous stratified epithelial layer. Derived from this conclusion is the fact that vaginal pathologies, progestogenic hormones or microbicides that decrease the thickness of the epithelium will increase the chances of HIV to meet its target cells. The increased number of monkeys vaginally infected with SIV/SHIV when pre-treated with DMPA clearly supports this contention. An inflammatory reaction recruiting target cells to the epithelium or the partial de-epithelialization and disruption of epithelial permeability caused by surfactants like nonoxynol-9 would have the same effect.

#### Emerging microbicide candidates

In his contribution to the "Drug Discovery" symposium, Martin Springer indicated that there was a significant attrition of candidate compounds from discovery to phase III clinical trials. Contrary to popular belief, most of this attrition is due to lack of sufficient efficacy. A plenary lecture by Charles Kelly provided an update of current and emerging microbicides. The most promising microbicide candidates in the pipeline belong to the mechanistic categories of reverse transcriptase inhibitors (RTIs), entry inhibitors (EIs) and integrase inhibitors (INIs). Within the RTIs, the most advanced compounds are tenofovir, dapirivine, UC-781 and MIV-150. Among EIs, there are CCR5 blockers (e.g., Maraviroc, RANTES analogs, M-167), gp-120 blockers (e.g., BMS-793), fusion inhibitors (e.g., T-1249) and a CD4 downmodulator (CADA). Two new fully recombinant RANTES analogs (peptides) were shown to prevent systemic viremia in 100% (5/5) of the monkeys challenged intravaginally with 300 TCID_50 _SHIV SF162P [5]. Mohammed Saifuddin reported a similar outcome for cellulose sulfate (CS) using a low-dose multiple-challenge X4/R5 SHIV model. Although most of the CS treated animals showed HIV positive proviral DNA and HIV-specific T cell responses, none of them seroconverted, showed systemic viremia (vRNA) or produced culturable virus.

In a symposium on "New Approaches", Robert Buckheit outlined the significance of pyrimidinediones, a new series of small molecules with anti-HIV activity in the subnanomolar range and a dual mechanism of action inhibiting RT and cell entry.

Combination microbicides were cited as a logical next step in microbicide development. Their advantages, as discussed in a symposium on "Combination Microbicides", include wider antiviral spectrum, higher genetic barrier to resistance, higher potency, and broader cell/tissue coverage. Combinations of RTIs such as tenofovir with TMC-120 or UC-781 are being explored and developed. Studies from different labs have shown that these combinations display synergistic activity (CI ~ 0.7) and are more potent and more active against resistant virus than their single ingredients alone [6,7].

Speaking on the induction of resistance, John Mellors stated that in spite of the valid concern about compounds generating and being susceptible to resistant viruses, product development emphasis should be put on potency and efficacy of single-active and combination microbicides. The impact of viral resistance in the context of microbicide use is significantly lower than that of resistance generated by antiviral therapy and is even smaller in the presence of reduced number of infections due the use of an effective microbicide.

Various delivery systems and dosage forms for combination microbicides are currently in development, as described by Patrick Kiser, with gels and two types of intravaginal rings being the most advanced systems. In addition to developmental challenges, combination microbicides also face an unexplored regulatory pathway.

#### Biomarkers, models and microbicide preclinical evaluation

In a panel discussion, Gustavo Doncel proposed a new preclinical testing algorithm for the rational selection of microbicide candidates. Three main parameters, efficacy, safety and pharmacokinetics and pharmacodynamics (PK/PD), are to be assessed sequentially in vitro, ex vivo (explants) and in vivo (animals). The need to incorporate environmental factors (e.g., pH, seminal plasma, cervicovaginal secretions and microflora) in these studies was highlighted by Raina Fichorova, Ken Rosenthal, and Radiana Trifonova in two symposia and one oral presentation [8], respectively. Improvements and development of new animal models were reported. Dorothy Patton [9] and Cecilia Cheng-Meyer described the use of single- and multiple-dose non-human primate models of HIV vaginal transmission to assess microbicide safety and efficacy (roundtable discussion on "Critical gaps in microbicide development"). Furthermore, cell-associated transmission of SIV is being optimized in a single-dose model by Roger Le Grand and colleagues.

A new humanized mouse model based on transplantation of human fetal bone marrow, liver and thymus into a SCID/NOD mouse has been shown by Victor Garcia and colleagues to be a suitable model for intravaginal HIV infection  [10]. If validated for microbicide testing, this model will be useful in early assessments of compound efficacy.

Epithelial integrity, permeability and overall barrier function must be evaluated in vitro, and, if possible, in vivo, when determining the profile of a compound in regard to cervicovaginal safety. Betsy Herold proposed that inflammatory mediators, immune innate factors and antimicrobial activity also be critical endpoints of preclinical safety evaluation.

Given the results of recent microbicide clinical effectiveness trials, both Gustavo Doncel and Marla Keller cited the importance of considering evaluating the potential of compounds and formulations to increase or enhance susceptibility to infection. In vitro and animal models already in existence or in development to assess this property [11] were described during a roundtable discussion by Marla Keller. For the most part they consist of pre-exposing tissues or animals (e.g., mice or monkeys) to repeated doses of microbicides before challenging them with a suboptimal infectious dose of virus in the absence of microbicide.

In the panel discussion on "Criteria to enable criticial selection of microbicides", Joe Romano emphasized that pharmacokinetic and, whenever possible, pharmacodynamic studies, both in vitro and in vivo, are crucial to understand how microbicides work at the tissue/cell level. Innovative methodology is being developed to assess these parameters, both clinically and preclinically.

The overall conclusion of the panel was that the rational selection of microbicide candidates for further clinical testing is a complex, iterative, evolving process that may be improved by harmonizing/standardizing a minimum set of assays and models, while preserving the ability of scientists to innovate. With the addition of new, more predictive biomarkers and models of safety and efficacy, a final Go/No Go decision should be based on a comprehensive preclinical package, rather than on a single "gatekeeper" assay or model.

#### Microbicide formulations and delivery systems

Formulations can "make or break" a microbicide product. For instance, in his plenary lecture Charles Lacey pointed out that a gel containing high concentrations of glycerol produced unacceptable adverse events in a phase I clinical trial. The hyperosmolarity of a product has been blamed for significant negative effects on mucosal tissue, especially that of the rectum [12]. Formulation properties can also be used to enhance the antiviral activity of microbicides [13]. Research and development of new delivery systems and dosage forms for vaginal and rectal microbicides is ongoing. Intravaginal rings were described as especially suitable for long-term controlled-release of hydrophobic compounds in two different symposia by Karl Malcolm and Patrick Kiser. Quick-dissolve thin films and tablets are also being investigated and developed to deliver microbicides, as described by Lisa Rohan and Sanjay Garg, respectively, in the symposium on "Dosage Forms for Microbicides."

Although more research is needed to determine the acceptability of different dosage forms, it is clear that cultural differences exist that may affect adherence to product use. Since the type and characteristics of formulations and delivery systems influence preclinical testing, clinical trial design and, ultimately, user acceptability, formulations and delivery systems should be designed taking all these parameters into consideration. Furthermore, they should be characterized and established as early as possible in the product development process.

### Track B – Clinical science

#### Sanjay Mehendale

Within this track there were two plenary sessions, one with Sharon Hillier providing an update on clinical trials of microbicide effectiveness and a second with Charles Lacey providing an overview of how product characteristics and delivery mechanisms may influence clinical trial design. Sharon Hillier provided an overview of current and future research products and some of the challenges we face in the conduct of clinical trials. The new generation of ARV-containing products were introduced with some information on planned trials.

General sessions were grouped into themes of 1) Update on clinical trials 2) statistics, power and design of clinical trials 3) studies in men 4) findings from early phases of clinical trials 5) impact of other prevention trials 6) clinical safety studies of vaginal microbicides and 7) lessons learnt from cohort studies. In addition there were cross track sessions on "when clinical trials end: challenges and lessons learned", and "standards of care". The sessions reported here have been divided into seven areas: Current status and findings from phase III trials: products in the advanced stage of evaluation; early safety trials; methodological issues in the design and implementation of clinical trials; impact of other HIV prevention trials; clinical safety studies; and when clinical trials end: challenges, experiences and lessons learned; and a short report on cross track sessions.

#### Current status and findings from phase III trials: Products in the advanced stage of evaluation

##### HPTN 035

HPTN 035 is a trial funded by the NIH to assess the effectiveness of PRO 2000 and BufferGel in preventing HIV infection in a cohort of women from South Africa, Malawi, Zimbabwe and Zambia. A total of 3200 women have been enrolled and are due to complete the study by August 2008. The study is designed to have two microbicide arms and two control arms (placebo and condom only). The results are expected in early 2009 [14].

##### MDP 301

The microbicide development program (MDP) is a collaboration between scientists in the UK (Imperial college and Clinical trials Unit of the Medical Research Council) and scientists from Uganda, Tanzania, Zambia and South Africa. The trial is being conducted in Africa [15]. The trial was initially testing two concentrations of PRO 2000 (0.5% and 2%) against a placebo. However in early February, the data and safety monitoring committee (DSMC) reviewed the data to find that the 2% PRO 2000 had very little chance of showing effectiveness. The 2% arm of the study was subsequently dropped. The study continues to enrol women on the 0.5% and placebo arms of the study and is expected to complete enrolment in August 2008. The results are expected in late 2009. Henry Luwugge reported that gel use appeared to be high irrespective of condom use [16]. Given the recent failures of products with similar modes of action, it is encouraging that to date major safety concerns have not been raised through several interim analysis in both the above ongoing trials.

##### Carraguard™

A plenary presentation by Elof Johannson provided the results of the Carraguard™ trial. Carraguard™, a sea weed extract, was the Population Council's lead microbicide product. A large phase III clinical trial involving 6202 women was conducted at 3 sites in South Africa; Durban, Shoshuguwe (Pretoria) and Cape Town. The results showed that although Carraguard™ was safe, it was not effective in preventing HIV transmission. The results were extremely disappointing [17].

The report of a sub study presented by Marlena Gehret to assess the association between male circumcision and partner sero-conversion was studied in 4579 women with a single partner [18]. Women with partners who were circumcised were more likely to have a lower HIV incidence. Thesla Palanee reported that incidence of STI among participants of Carraguard™ trial showed a high prevalence of *C. trachomatis *(CT) and *T. vaginalis *(TV) infection [19]. The prevalence rates varied across the three African sites. At follow-up, decreasing trends in the incidence of STI was observed. This could be due to counseling, improved health seeking behavior and STI treatment. Risk factors associated with HIV incidence included number of partners, and being positive for CT, TV and bacterial vaginosis (BV).

Results of another study of the impact of behavioural change on STI incidence by Felicity Gopolang showed that there was a reduction in the number of sex acts, number of partners and increase in condom use [20]. However, the observed behavioral change had no impact on the overall STI incidence rate. Adherence to product use was measured through the use of biomarkers, self-report and a combination of both methods. Nearly 96% of the 6005 women self-reported adherence to the product. Gel used during vaginal sex was 57%, condom use 64%, both gel and condom use 62%; women reported having sex 2.5 times per week and applicator insertion 0.9 times per week. Analysis of self-reported condom use and microscopy for sperms in vaginal swabs (wet mount and TV In pouch) showed detectable spermatozoa among that reporting condom use in 62.5%. This study indicated that information given by women on condom use may not be reliable.

##### CAPRISA 004

In addition to trials of current generation of microbicides, an update was provided on the first proof of concept ARV-based microbicide trial being conducted in South Africa. CAPRISA 004 is a two arm study comparing a coitally dependent dose of 1% tenofovir against a placebo. Enrollment is scheduled to be completed by July and results expected in 2010 [21].

##### Savvy

The vaginal microbicide Savvy was evaluated in a Phase III Nigerian trial which required the use of pre-loaded single use gel applicators and a placebo, and enrolled 2142 women. The trial was terminated early at 75% completion level. High pregnancy rates were observed across both study arms. There was no evidence of the effectiveness of the product in preventing HIV infection. Paul Feldblum concluded that more conservative HIV incidence estimations should be employed and more effective family planning measures should be advocated among the trial participants [22].

#### Early safety trials

##### HPTN 059

Sharon Hillier presented some very encouraging results of the safety and coitally dependent use of 1% tenofovir over six months [23]. The HPTN 059 trial also tested the safety and acceptability of 1% Tenofovir gel and was conducted in India and two US sites [24]. Soma Das reported 96% retention over 6 months and 80–85% product adherence. Preliminary results indicate that the safety profile of the product was excellent and toxicity end points were comparable in product and placebo arms. There were no significant sexually transmitted infection (STI) acquisitions. Daily gel use was close to 20% and over 75% with sex. Some tenofovir was detectable in blood in 75% of those who had used gel in the past 24 hours. Strategies such as locator forms, participant tracking database, telephonic contacts, home visits, clinic diary and follow-up schedule chart were used to facilitate retention. Male involvement and proper scheduling helped in achieving remarkably high retention rate in this study.

In a study presented by Jill Schwartz, two 1% tenofovir gel dosing regimens are currently being evaluated in pharmacokinetic studies on samples collected by cytobrush and biopsies from the posterior fornix. Advanced assays are being used for local and systemic compartment evaluations.

##### Dapivirine vaginal gel (Gel-002)

Gel-002 with active ingredient Dapivirine recently underwent safety and tolerability testing in studies in Rwanda, South Africa and Tanzania. This NNRTI was tested in a gel form to assess its safety and acceptability. Shanique Smythe presented the data from this 3 dose study requiring 42 days' product use. The product was shown to be safe and well-tolerated, possibly warranting further investigation of the gel [25]. Annalene Nel presented the results of a South African phase I pharmacokinetic study of Gel-002 among 18 women in 3 groups. Low systemic levels of the product were detected up to 24 hours after exposure [26].

##### Cellulose acetate phthalate (CAP 13% gel)

Charles Lacey presented a Phase I study of this hyper-osmolar vaginal gel in 10 women which was stopped following unacceptable adverse events. A continuous, heavy and watery discharge was noted in half of the women participating in the study [27].

##### Vivagel

Vivagel (SPL7013 Gel), a product with anti-HIV and anti-herpes properties, was initially tested in macaque models and subsequently in two studies in men and women. Clare Price and Maureen Momanyi reported findings from Vivagel studies. Safety after penile application of 2 mg of product for 7 days showed no genital irritation, genital pruritus, penile dryness or scrotal tingling and the results were similar in circumcised and uncircumcised men. The product was found to be safe and well tolerated. The acceptability was good and there was no evidence of systemic absorption [28,29].

##### Special cross track sessions: Key-note addresses, symposia and panel discussions

Within track B three symposia were organized on "Rectal Microbicides", "Moving Microbicides into susceptible populations" (discussed under track D) and "Microbicides and HIV positive women" (discussed under track D) and two key-note addresses on "Methodological issues in design and implementation of clinical trials". Additionally, two panel discussions were conducted on the "Impact of other HIV prevention trials" and "Clinical safety studies". The major observations during these sessions are summarized below.

### Studies on rectal exposures of microbicides

An ex-vivo challenge study of the safety of the rectal microbicide UC-781 on explants has been completed and the data is still blinded. A subsequent interim report of the safety study at 50% completion was presented by Peter Anton [30]. Interim data from 36 rectally-exposed men and women at UCLA, USA indicated that the product was safe and well-tolerated. Product adherence was good and no Grade III or IV adverse reactions were observed. Even at 75% study completion results remain unchanged. The study employed a vaginal applicator for rectal use. Further research needs to be conducted on the design and acceptability of the vaginal applicator for rectal use. Mucosal immunity studies following two weekly applications for 6 weeks indicated no changes in the mucosal and cytokine profiles of the three groups over time.

### Methodological issues in the design and implementation of clinical trials

Doug Taylor gave a very informative talk on statistical power. He expressed the challenges we face in assessing trial outcomes on factors that impact on power. There are several factors that contribute to the power of the study to show efficacy. These include high retention rates, adherence to product use, women taken off the product due to pregnancy and high incidence rates. He expressed the importance of conducting effectiveness trials over efficacy trials. To overcome the challenges of high incidence of pregnancy and its impact on power, new generation of products will be tested for safety during pregnancy.

Zeda Rosenberg discussed the challenges in doing phase III studies with enough power when a product is believed to be effective in preventing HIV transmission. She explained that the current trials do not measure efficacy, but it is inferred that a woman has become infected after unknown number of sex acts. She stressed that effectiveness trials are more important because they measure both efficacy as well as adherence. It is being increasingly felt that alternative study designs should be employed for the fast tracking of microbicide development and testing. The best among the next generation products must be selected for safety evaluation and should be evaluated early for futility after employing measures to improve adherence. They should be powered for licensure. Alternate studies of superior design will allow for improved ways of measuring adherence and reductions of risk taking behaviour, and at the same time test the effectiveness of the product in HIV prevention.

### Impact of other HIV prevention trials

Globally the agenda of HIV prevention research includes behavioral change, circumcision, diaphragm use, pre-exposure prophylaxis and STI control. We have learned that adherence significantly impacts the outcomes of trials. Trials have also had setbacks due to unexpected fewer number of study end-points resulting from low HIV incidence. According to Lut van Damme and colleagues, lower than expected HIV incidence rates highlight the importance of site preparatory studies and newer technologies to estimate HIV incidence.

### HSV-2 suppressive therapy for HIV prevention

The HPTN 039 study, conducted by Connie Celum, explored whether HSV-2 suppression by Acyclovir could reduce the risk of HIV acquisition [31]. Although the precise reasons why this trial failed to show the desired result in Africa are unknown, the lack of adherence to Acyclovir prophylaxis may appear to be one of the reasons. This is the second trial of Acyclovir which failed to show effectiveness in preventing HIV. A trial conducted in Tanzania showed similar results [32].

### Male circumcision

Cate Hankins presented the outcome of the three trials on male circumcision in the prevention of HIV. The results showed the male circumcision provided up to 60% protection against female to male transmission of HIV. The current challenges were to roll out male circumcision as an HIV prevention option in developing countries.

### Vaginal diaphragm for the prevention of HIV

MIRA (Methods of Improving reproductive Health in Africa) was a trial conducted to assess the effectiveness of the vaginal diaphragm in the prevention of HIV. The trial was completed in early 2007 and the results reported in July of the same year [2]. The study was conducted at 3 sites in Southern Africa; Zimbabwe and South Africa (Johannesburg and Durban). The study showed that the vaginal diaphragm did not provide any added benefit when used in addition to the current prevention package of male condoms, risk reduction counseling and treatment for STI. Kelly Blanchard presented an overview of the trial and the results [33].

### Clinical safety studies

Clinical safety studies evaluate integrity of epithelium, effects on the penis, vaginal microflora, pregnancy and impact on body function due to systemic absorption or inflammatory response. Symptoms and colposcopy do not necessarily indicate safety and recent outcomes of trials suggest that there is a need to identify markers for general toxicity at an early stage of clinical trials. It is essential to establish safety both in HIV negative and positive women. The possibility of extension of the product in endocervix and uterus warrants some method like Magnetic Resonance Imaging (MRI) for its safety evaluation, which might increase the cost of a trial substantially. Jonathan Weber emphasized the urgent need for long-term safety assessment and strategies to avoid products going into large scale trials and then showing increased safety concerns.

#### Cross track sessions

##### Standard of Care

In a cross track session on standard of care, Kathy Shapiro presented the results of a mapping exercise by the Global Campaign for Microbicides. Key conclusions from this study were that clinical trial sites are providing better quality (access, affordability, skills of providers) of services. Also there are a range of standards of care being offered at sites depending on public sector services in the area. She outlined recommendations that came out of the exercise and subsequent meetings; urging that communities should be engaged in decisions about how to manage trial standard of care; future trials should consider co-locating with existing local care facilities and use the opportunity to improve the services provided, improve the quality of care and build the capacity of health care providers; trial sites should also improve their referral systems and facilitate women's access to care at referral facilities; and as much as possible to also establish formal agreements with referral sites to avoid the unnecessary repetition of tests and other procedures.

##### When clinical trials end: Challenges, experiences and lessons learned

Three speakers in this session, Lut Van Damme, Gita Ramjee and Manju Chatani provided their perspectives on the impact of the unexpected closure of clinical trials mainly due to safety concerns. Lut Van Damme provided an overview of the challenges faced by sponsors during the closure of Cellulose Sulphate trial in early 2007. Key lessons learned were the need for communication, and visiting trial sites immediately. She stressed that one is never prepared emotionally and practically for sudden closure of trials.

Manju Chatani's contribution to a panel discussion outlined a number of lessons learned from the advocates' perspective after the closure and premature end of microbicide trials. These include; the need to strengthen communication strategies in the microbicides field, the need to select effective community speakers and inform them routinely on developments and be informed from them as well and the need to conduct ongoing media training for these spokesperson. A key lesson is to work closely with media agencies on an ongoing basis – so that media is well-versed with protocols of microbicides research and is an effective partner. There is a value in scenario planning in anticipation of different possible results. These experiences have led to advocates having increased access to researchers and information, and as a result there is more coordination and collaboration among these stakeholders.

The MMCI completed a case study of lessons learned during the unexpected closures of the Cellulose Sulfate trial and the inflammatory press coverage that followed in South Africa. The case study analyzes the handling of the crisis in light of conflicting pressures at the international, local, and trial site level. It highlights the particular challenges of ensuring that relevant stakeholders and trial participants are informed of the findings before they are released to the media.

Gita Ramjee gave her perspective as an investigator at trial sites. Many of the sites employ skilled staff to conduct clinical trials. When trials end either prematurely or due to natural completion, it is difficult to sustain trial staff if no other products are available to go into large scale trials. This poses a huge challenge for trial sites. Unexpected closures have been challenging due to sensationalist articles in the media that impact on HIV prevention efforts. Effective communication and scenario planning of trial outcomes need to be in place well in advance of the trial outcomes. She stressed the involvement of the community in the dissemination of results and preparation of results in local language. All speakers stressed the importance of communication and collaboration with various disciplines prior to release of the results.

In addition to the cross track sessions there were special symposia on microbicides and HIV positive women. The symposium presenters were Wafaa EL Sader and Anna Forbes. Both presenters stressed the importance of involving HIV positive women in HIV prevention efforts. The second symposium was on moving microbicide research to vulnerable populations. Richard Beigi presented on the need for involving pregnant women in clinical trials to assess the safety of products. Ian McGowan stressed the importance of involving men who have sex with men and development of rectal microbicides. Kathy Slack highlighted the growing incidence of HIV in adolescents and the need to involve adolescents in clinical trials of microbicides and other prevention technologies.

### Track C – Behavioural and social science

#### Elizabeth E. Tolley

The social science Track C program featured a wide range of research topics and methodological approaches. Seven abstract-driven sessions and three invited presentations addressed issues pertaining to adherence; behavioural and social science methods and tools; acceptability; community involvement; partner roles; vulnerable populations; and future access.

#### Cultural influence

In his plenary talk, Ravi Varma described the ways that culture influences men's and women's risk through gender roles and relationships – and will ultimately determine whether, with whom and how gels may be used. Some men's first sexual encounters were with other men; many men who engage in sex with other men are married. Men's perception of risk from such behaviours was low – especially if one was the "penetrator". Men who engaged in extramarital sex (whether with other women or men) were six times more likely to report wife abuse than those who did not. Yet, gender norms perpetuate women's submission to coercive sex in marriage and prevent frank discussions about sexuality and risk. Varma concluded that while the introduction of microbicides may act as a catalyst, more encompassing gender transformative strategies were needed to reduce men's and women's risk of HIV in India. Indeed, the association between microbicide introduction and gender norm transformation arose in South Africa as well, where Fern Terris-Prestholt found a preference for promotion of microbicides as a means to empower women and/or prevent HIV – rather than a method to enhance sexual pleasure, in a survey of 1017 women in three townships in Johannesburg, South Africa [34].

Several studies reminded us that the cultural context of microbicide use may vary across countries and for different groups within a population. This included access to partially effective microbicides and its impact on vulnerability of sex workers [35], types of products, frequency and timing of use [36] and modelling studies to ascertain impact of microbicide introduction [37].

#### Communities

Communities are not homogenous. Multiple stakeholders, including civil society groups [38], healthcare providers [39], clinical trial staff [40] and trial participants influence trial acceptability through their understanding and expectations of clinical trials. One important factor influencing successful trial implementation is community perception of the quality and types of HIV-related treatment, care and support to be provided to those who screen out of the trial or sero-convert, their families or the broader community. Community involvement and partnership is critical to initiation and completion of trials to ensure such concerns are adequately addressed. The benefit of such involvement was evident in premature closure of the Cellulose Sulphate trial [41], where intensive counselling, community education and on-going informed consent processes led to relatively positive community reactions despite a spate of negative and inaccurate publicity about closure. While there are cultural differences, trial sites have to develop novel strategies to explain the understanding of trial methodologies such as randomization and placebo [42,40]. This requires ongoing improvement of the informed consent process and understanding. In addition it was pointed out that communities should have a sense of ownership for successful community involvement. New trials will implement comprehensive socio-behavioural and community preparedness activities to allow for community partnerships [38,43].

#### Trials and clinic settings

The clinical trial setting itself is likely to influence individuals' risk behaviours; several studies examined changes in trial participants' behaviours post randomization. Among 2140 women randomized to the diaphragm arm of the MIRA trial, just over 22% reported using diaphragms instead of condoms at all visits, and over 60% reported doing so at some visits [2,33]. Women who consistently reported substituting the diaphragm for condoms (versus never report this behaviour) were more likely to be engaged in sex work, report domestic violence or sex with a drunken partner, believe that diaphragms protect against HIV/STIs, and are less likely to know that condoms can be used with diaphragms.

While Ariane Van der Straten's study identified the potential for therapeutic misconception and condom migration, Adelaide Mzimela's and Elizabeth Tolley's studies found that trial participation may, in fact, lead to higher use of condoms [44,45].

#### Partners

Women's ability to negotiate trial participation with intimate partners may determine who enrolls in trials and how well they are able to adhere to gel and other trial requirements. Anna Dladla-Qwabe examined the relationship between consistent gel adherence and disclosure to partners of study participation/gel use in a study of 263 women in Hlabisa, South Africa participating in the HPTN 035 trial [46]. She noted that women reporting consistent gel use were more likely to have disclosed both their study participation and their use of gel to partners than women who reported inconsistent use. Local behavioural investigators at several MDP trial sites examined the role of men in gel use. Disclosure to gel use was reported by many participants in several trials and, in general, men supported women's use of the product. Lubrication provided by the gel increased sexual pleasure hence the products were acceptable [47-51].

On the other hand Petina Musara examined covert use of microbicides in a sub-study of HPTN 035 in Zimbabwe [52]. Women reported a motivation to use microbicides clandestinely when they suspected a partner to be unfaithful; when he was unwilling to use condoms; or when he was HIV positive. However, requirements for timing and insertion of gel, as well as difficulties of storage limited covert use. Community leaders also expressed negative opinions about covert use, further constraining this approach.

#### Participants

Several Track C presentations identified individual-level factors as influencing acceptability and use of microbicides. These included perceptions of product attributes, perception of risk and efficacy to communicate with a partner about risk and/or negotiate risk reduction behaviours. For example, in PRO 2000/5 study of 3157 women, Jessica Dhookie noted very few reports of difficulty with insertion (> 1%) – all reported in week 4 follow-up only [50]. Alex Carballo-Diéguez examined the acceptability of rectal microbicide vehicle-related attributes in a cross-over trial of 77 HIV-negative cohort of men having sex with men [53]. Men and their partners were significantly more likely to prefer gel over suppositories, reporting that gel was associated with greater sexual satisfaction and resulted in less leakage, bloating or other side effects than did suppositories. In a cross-sectional U.S.-based study called the Phoenix Project, Kate Morrow found that a person's attitudes and characterizations of risk were influenced by specific partner contexts and that willingness to use a microbicide was affected by both those individual and relationship elements [54]. In India, Elizabeth Tolley found somewhat similar measures of risk perception and partner context, as well as product attitudes and perception of protection efficacy, to predict consistency of condom use among clinical trial participants, but only product attitudes to predict gel use [45].

While several presentations focused on the individual, couple-related or other factors that might explain participants' level of adherence in trials, only one presentation focused on approaches to optimizing participants' adherence within trials. Approaches include motivational interviewing to assist individual participants in identifying and pre-empting situations that could lead to non-adherence of gel [55].

#### Inside the body

Finally, in her keynote address, Kate Morrow described her collaboration with basic scientists. As they work to optimize microbicide gel deployment, her aim is to adequately measure individual perceptions' of deployment (or other product properties) and link them to biophysical characteristics – so that such characteristics can be assessed by the user. Such a linkage would enable product developers to more rationally select formulations that have a greater chance of success – because participants may be more adherent.

#### Cross-cutting themes

An important cross-cutting theme for the Microbicide 2008 Track C program was the need for social scientists to pay more attention to measurement. Topics pertaining to measurement included 1) the development and use of psychometric scales; 2) triangulation of information through mixed method approaches; and 3) assessment of different data collection strategies. In her keynote address, Geraldine Barrett described her work to develop a more valid and reliable measure of unplanned pregnancy for use in the United Kingdom. Barrett relied on a two step process, first using qualitative methods to identify and define key factors associated with women's attitudes towards a recent pregnancy; and then the use of quantitative methods to develop a six item psychometric scale that more accurately captures the range of positions women might have towards pregnancy. Kate Morrow used a similar process of qualitative research and psychometric scale development to behavioural tools that better assess women's perception of how microbicide products perform within the body.

In addition to improving measures for specific concepts through psychometric scale development, Robert Pool provided examples of how the use of multiple data collection methods could improve understanding and ultimately the accuracy of data on adherence [56]. Ana Ventuneac described the advantages and shortcomings of three data collection strategies: 1) interactive voice response systems; 2) web-based and computer assisted self-interviews; and 3) use of hand-held devices [57]. Such approaches can increase privacy and frequency of data collection, thereby decreasing social desirability and recall biases that lead to inaccurate reporting.

A second cross-cutting theme was the challenge of implementing behavioural and social science research in microbicide trials and the need for greater collaboration and networking within these disciplines [58].

### Track D – Policy, advocacy and community

#### Kim Dickson

Track D highlighted the issues of policy, advocacy and community relating to microbicides research, development, access and introduction. The Track D presentations and discussions revolved around the key themes of community involvement; managing stakeholders expectations; modelling the impact of microbicides introduction; engaging a broader audience in microbicides advocacy; and other key issues.

#### Community involvement

A keynote talk by Mangala Patil on defining the community and the importance of partnerships for HIV research emphasized how the community advisory board (CAB) has an important role to play to be the bridge between trial participants, the broader community in the region, and researchers, scientists and clinicians. Another key role of the CAB was to communicate effectively to dispel misconceptions or myths that participants or the trial community might have.

The presentations on community involvement highlighted how community engagement has become more sophisticated with more innovative strategies being employed. These strategies included the use toll free lines, homes visits and peer educators. They outlined how civil society engagement helps microbicide trials in many ways, including: helping research to avoid pitfalls, maintaining accountability between researchers and community, mobilizing new public resources, facilitating open and fair communication between different stakeholders thereby increasing accountability and increasing trust in the communities. The presentations reinforced the importance of assessing the needs of the community and involving them in all stages of research.

Although advocates continue to request for early community engagement in the protocol development process, including the concept development stage many researchers feel this is difficult. Morenike Ukpong presented new strategies to involve community at all levels [59].

A presentation by Majorie Nakimuli [60] outlined the effectiveness of peer leaders in Uganda from the Makere/Mulago/CONRAD Center project in the mobilization, recruitment and retention of participants. These peer leaders worked alongside the CAB and belonged to the community where trial participants were from. They were crucial in mobilizing the community through training and leadership [61].

Anna Forbes explained the process the Global Campaign for Microbicides spearheaded to identify seven key gaps in civil society involvement throughout the microbicide research and development and post-development process and make recommendations for seven key priority actions for how funders, researchers, governments, and civil society working together could address each of these gaps [62]. The Microbicide Development Strategy (MDS) also proposes a funding mechanism to donor agencies to provide a window to key civil society organizations working on microbicides research, advocacy and development.

#### Managing stakeholder expectations of trial results

Presentations in these sessions emphasized the importance of preparing for communications and unexpected results and the need to understand how to frame data to meet the needs of different audiences including advocates, policy makers, providers, participants and communities.

A presentation from the Microbicides Media Initiative (MMCI) by Deborah Baron emphasized that developing clear, concise and consistent messages on interpreting trial results has become a top priority for the microbicides field whether studies are halted prematurely or finish on schedule [63]. The Microbicides Media and Communication Initiative (MMCI) was launched in 2005 to help the wider microbicide field anticipate and respond proactively to the communications challenges posed by large-scale trials in Africa and Asia. Since then, the MMCI has become a forum for communications staff, researchers and advocates to share expertise, resources and ideas across institutions and networks.

#### Modelling the impact of microbicides introduction

Charlotte Watts used mathematical modelling to explore the relationship between 'efficacy' and 'effectiveness' measures [64]. Phase III microbicide trials provide the strongest evidence about HIV impact. For coitally dependent products, inevitably the measure of trial effectiveness will be lower than the per sex act protection provided, with greater differences for lower consistency use. Estimates of contraceptive efficacy come from prospective observational studies comparing users with non-users. Non-user dependent methods are > 96% effective, with rates less than 80% being viewed as inadequate. Estimates of condom HIV efficacy come from comparisons of infection rates among discordant couples with different reported levels of condom use, with meta analysis suggesting that consistent use reduces cumulative risk by 87%, and inconsistent use by 60%. The 90% – 95% condom efficacy term refers to per sex act reduction in risk, and is derived from modelling. Impact of microbicides is different on: risk per sex act, on individual risk if used overtime, on HIV incidence in a controlled trial setting and on population HIV incidence following widespread provision. The presentation concluded that the findings highlight the need for clarity in the way in which the terms 'efficacy' and 'effectiveness' are used across fields, and for care when communicating and interpreting trial results and that we need to find better ways of communicating the results to different stakeholders.

An keynote presentation by Sally Blower on modelling the use of rectal microbicides in 'bathouses' concluded that moderately effective rectal microbicides (50% efficacy) with moderate use (30% use) could have substantial impact on HIV prevention in these bathouses. Blower highlighted the impact of anti retroviral based (ARV) based microbicides derived from modelling studies. She concluded that microbicides are important empowerment tools for women but paradoxically ARV based-microbicides could benefit men more than women in terms of infection prevented and infections prevented per resistant cases, especially if the microbicide was 'high-risk' (in terms of the possibility for acquiring resistance).

#### Engaging a broader audience in microbicides advocacy

Presentations in these sessions focused on the issues of rectal microbicides advocacy reaching young women and addressing the issues of HIV positive women.

#### Rectal microbicides

A comprehensive advocacy approach, spanning science, policy and the community, is needed to bring safe, acceptable and effective rectal microbicides to market. James Pickett of the International Rectal Microbicides Working Group expressed that data consistently reveal that men and women around the world engage in receptive anal intercourse, often without the use of a condom [65]. He stressed that the development of a rectal microbicide, which could provide some protection from HIV transmission during anal intercourse, is hampered by biological and scientific challenges, socio-cultural and political barriers such as stigma, denial and homophobia, and a lack of adequate resources necessary for the development of a robust pipeline of rectal microbicide candidates. He called for rectal safety studies on all viable vaginal microbicides and stressed that testing of additional commercial lubricants for rectal safety be encouraged and regulatory bodies and policies should be supportive too. Nesha Haniff presented her experiences of rectal microbicides advocacy through the work of Jamaica Aids Support for Life [66].

### Moving microbicides into susceptible populations

#### Adolescents

The topic of adolescents was covered in both track B and D. Martha Brady highlighted the gap in policy and programmes to build the social and health platform for the adolescents (10–19 years), youth (15–24 years), especially young girls [67]. Conventional "youth programs" and "adolescent sexual reproductive health" programs do not reach the majority of vulnerable adolescent girls, who remain overlooked and underserved. Any effort to reach adolescents girls and young women with a microbicide product would need to address the heterogeneity of this potential user group: they have diverse needs and situations they deal with. Targeted messages and a range of protection strategies and services for a diverse group of girls will be needed.

#### Microbicides and HIV positive women

Sean Philpott, Louise Binder [68] and a roundtable panel of HIV positive women, researchers and advocates stressed the need to involve HIV positive women in microbicides research. They mentioned that as microbicides are being developed, HIV-positive women are raising numerous issues related to safety, trial design and ethics, which require consideration and implementation in current trials and future research. The roundtable discussions outlined how research with HIV positive women and engagement of HIV positive women in advocacy has increased. The discussions emphasized that communication channels to keep HIV positive women's groups apprised of new developments on microbicides research should be developed.

#### Other key issues

Helen Rees's insightful key note on lessons from experience with other reproductive health technologies stressed that introduction of new products and widespread access and use take a long time; it took more than 30 years for the tampon to gain widespread acceptability and use. Lessons drawn from the introduction of the Lippes Loop and Norplant have taught us to carefully evaluate service capability, the technology and consumer acceptability before introducing new technologies. Rees summarised key lessons from product introduction as; price; political context; distribution channel; marketing and targeting of advertising.

Panel sessions were held to discuss issues key issues including; 'Confronting the evidence' and planning for introduction and access. The 'evidence' discussion concluded that the level 'evidence' needed differs depending on the 'perceived' acceptability of the intervention, the researcher and complexity of implementation. Participants and communities need to be consulted about what evidence is acceptable and informed of new findings. The 'access and introduction' session revealed that; sponsors and trialists are working together and thinking about access after Phase III trials and are including cost, distribution and manufacturing in contracts to access their products for trials.

#### Satellite sessions and workshops

In addition to the full scientific programme, there were several satellite sessions. The NIH held a grantsmanship workshop for young investigators which included sessions on the components of a successful grant application, electronic submission tips, funding opportunities for international investigators and opportunities for collaboration.

There was a workshop for junior social scientists organized by the Office for AIDS Research (OAR) which covered a wide range of pertinent topics related to social science research within a clinical trial setting.

A pre-conference workshop on 'Health Advocacy" was co-hosted by AMAG, GCM, INN, NCHI and PWN. The workshop aimed to provide a status report on microbicide science and advocacy including the history of the field, a snapshot of the current status of the field, and discussion on some of the issues that participants were likely to raise.

The Alliance of Microbicide Development had a workshop on industrializing microbicides which covered a wide range of topics such as toxicology guidelines, manufacturing chemical and biological microbicides, intellectual property considerations and regulatory issues.

A symposium on rectal microbicides was co-hosted by AMFAR, UCLA AIDS Institute and the University of Pittsburgh. The objective was to provide an overview of recent advances in rectal microbicide development, improvement in applicator design and an update on advocacy directed towards increasing funding for rectal microbicide research.

Finally, there was a workshop on ARV and ARV resistance hosted by NIH/OAR. Given the importance of the topic with recent advances in microbicide and PreP (Pre-exposure Prophylaxis) trials, this workshop covered an overview of the microbicide pipeline, description of ARV resistance and the impact of potential resistance on participants, and some of the key challenges in ARV-based microbicide clinical trials.

## Summary

The Microbicides 2008 Conference in New Delhi was a resounding success. Despite the recent set backs in the HIV prevention field, with many products showing lack of effectiveness in preventing HIV, the scientists at the conference showed progress in the field of basic science. Valuable lessons learned from previous trials across all disciplines from basic and social sciences, as well as community involvement and advocacy, have greatly improved our understanding of the complexity of conducting HIV prevention research in a resource poor setting. Scientists reaffirmed their commitment to a continued search for a women-initiated HIV prevention option. The next meeting, planned for 2010, will be held in Pittsburgh, USA. The conference will be chaired by Sharon Hillier, Ian McGowan, and Gita Ramjee.

## Competing interests

The authors declare that they have no competing interests.

## Authors' contributions

GR co-chaired the Microbicides 2008 conference, invited author contributions for the various track summaries, provided summaries of general sessions and workshops, and edited this manuscript. Summaries of conference tracks A, B, C, and D, where written by GFD, SM, EET, and KD, respectively. All authors read and approved the final manuscript.
